# Community and Social Context: An Important Social Determinant of Cardiovascular Disease

**DOI:** 10.14797/mdcvj.846

**Published:** 2021-09-24

**Authors:** Rahul Singh, Zulqarnain Javed, Tamer Yahya, Javier Valero-Elizondo, Isaac Acquah, Adnan A. Hyder, Muhammad Haisum Maqsood, Zahir Amin, Sadeer Al-Kindi, Miguel Cainzos-Achirica, Khurram Nasir

**Affiliations:** 1 Department of Cardiology, University of Minnesota, Minnesota, US; 2 Division of Health Equity & Disparities Research, Center for Outcomes Research, Houston Methodist, Houston, Texas, US; 3 Center for Outcomes Research, Houston Methodist, Houston, Texas, US; 4 Houston Methodist DeBakey Heart & Vascular Center, Houston, Texas, USA; 5 Center for Cardiovascular Computational Health & Precision Medicine (C3-PH), Houston Methodist, Houston, Texas, US; 6 George Washington University, Washington, DC, US; 7 Lincoln Medical Center, New York, New York, US; 8 University of Houston, Houston, Texas, US; 9 Case Western Reserve University School of Medicine, Cleveland, Ohio, US

**Keywords:** community, social context, cardiovascular disease, social determinants of health

## Abstract

Disease prevention frameworks and clinical practice guidelines in the United States (US) have traditionally ignored upstream social determinants of health (SDOH), which are critical for reducing disparities in cardiovascular disease (CVD)—the leading cause of death in the US. Existing evidence demonstrates a protective effect of social support, social cohesion, and community engagement on overall health and wellbeing. Increasing community and social support is a major objective of the Healthy People 2030 initiative, with special provisions for vulnerable populations. However, to date, existing evidence of the association between community and social context (CSC)—an integral SDOH domain—and CVD has not been reviewed extensively. In particular, the individual and cumulative impact of CSC on CVD risk and the pathways linking CSC to cardiovascular outcomes are not well understood. In this review, we critically appraise current knowledge of the association between CSC and CVD, describe potential pathways linking CSC to CVD, and identify opportunities for evidence-based policy and practice interventions to improve CVD outcomes.

## Introduction

Cardiovascular disease (CVD) affects more than 480 million people annually worldwide.^1^ In the United States (US) alone, nearly 655,000 Americans die each year of CVD.^2^ It is known that traditional clinical risk factors such as diabetes, hypertension, and obesity, and modifiable risk behaviors including insufficient physical activity, poor diet, smoking, and alcohol consumption, account for over 80% of all CVD.^3^ Yet, most lifestyle CVD interventions focus on addressing downstream risk factors for disease, often failing to address the “causes of the causes.”^4,5^ Disease prevention frameworks and clinical practice guidelines have historically ignored *upstream* social determinants of health (SDOH), which are critical toward achieving primary prevention and reducing health disparities in CVD.^6,7^ In this context, a recent joint American College of Cardiology (ACC)/American Heart Association (AHA) clinical practice guideline emphasized the need to address SDOH to inform delivery of care and achieve primary prevention.^6^

Healthy People 2030 is a key initiative of the US Department of Health and Human Services’ Office of Disease Prevention and Health Promotion. Designed to improve the nation’s health and wellbeing, Healthy People 2030 sets forth specific objectives to create social and physical environments that help achieve optimal population health.^8^ Improved community and social support—a key SDOH—is a major objective, with special provisions for vulnerable populations including children/adolescents, racial/ethnic minorities and the lesbian, gay, bisexual, and transgender population.^8^ Existing evidence suggests a protective effect of social support, social cohesion, community engagement, and other community and social context (CSC) subdomains on overall health and wellbeing.^8,9^ However, relatively few studies have examined the impact of CSC on CVD risk or the possible pathways linking CSC to CVD outcomes, both of which merit further research. This review is intended to (1) critically appraise current knowledge of the association between CSC and CVD, (2) elucidate potential pathways and mechanisms through which CSC may predict adverse CVD outcomes, and (3) identify opportunities for evidence-based interventions to improve CVD outcomes and reduce disparities.

## Community and Social Context: An Integral Part of SDOH

**Figure 1:**
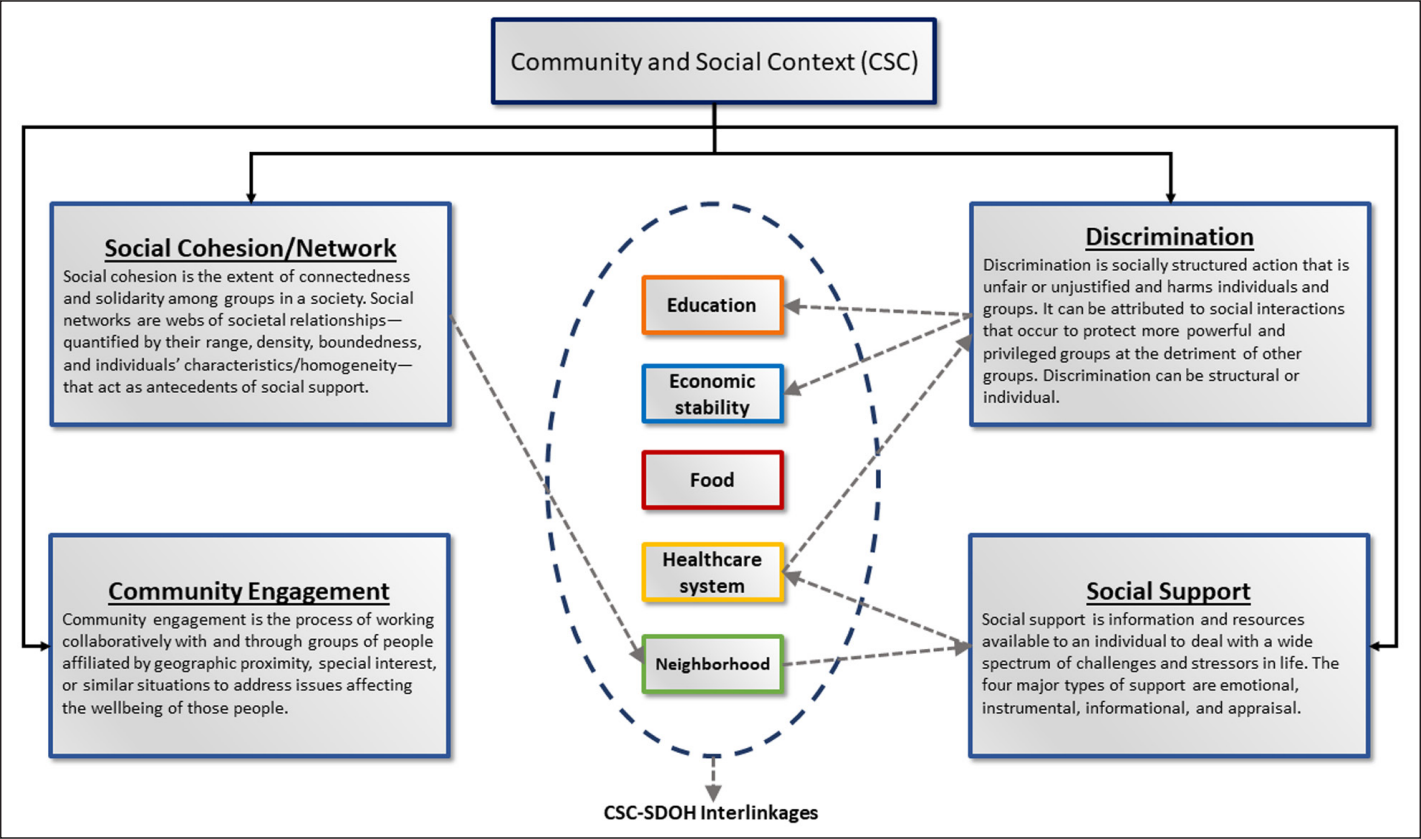
Community and social context: interlinkages with other social determinants of health (SDOH) domains.

Widely used SDOH models, such as the Healthy People and Kaiser Family Foundation models, provide critical domain-based frameworks for greater understanding of SDOH and design of evidence-based interventions to address SDOH.^8,10^ Community and social context is defined as “the context in which individual, societal, and cultural factors interact to impact health outcomes,”^11^ and it is an integral part of SDOH. SDOH are broadly classified into six major domains: economic stability, education, food, CSC, neighborhood and physical environment, and healthcare system.^8,10^ Each SDOH domain is linked to others via multiple pathways, with major CSC-SDOH interlinkages outlined in ***Figure 1***.

We identified four recurring themes in available literature on CSC and accordingly divided the domain into four distinct subdomains: social support, social cohesion/social networks, discrimination, and community engagement and civic participation (***Figure 1***).^10,11,12,13^ The following section discusses the impact of individual CSC subdomains on cardiovascular health using evidence from existing literature. Different measures used to define CSC subdomains, as reported in the literature and referenced herein, are listed in ***Table 1***.^14,15,16,17,18,19,20,21,22,23,24,25,26,27,28,29,30,31,32,33,34,35,36,37,38^

**Table 1:** Community and social context subdomain measures.^14,15,16,17,18,19,20,21,22,23,24,25,26,27,28,29,30,31,32,33,34,35,36,37,38^


STUDY	CITATION NUMBER	SUBDOMAIN DEFINITION/RELEVANT LINKS

**SOCIAL SUPPORT**		

Gallagher et al., 2011	14	Aspects of relationships with a partner that promote health or buffer stress including instrumental aid, emotional caring or concern, and information; final measure created using a survey questionnaire with multiple items *https://pubmed.ncbi.nlm.nih.gov/21372734/*

Wu et al., 2013	15	Perceived social support, using Multidimensional PerceivedSocial Support Scale *https://pubmed.ncbi.nlm.nih.gov/22746258/*

Kawachi et al., 1996	16	Berkman-Syme Social Networks Index: a composite measure of social connections. Major domains include marital status, sociability, church group membership, other community organization membership. *https://pubmed.ncbi.nlm.nih.gov/8935453/*

Berkman et al., 1992	17	Emotional support, measured using social ties (eg, can you count on anyone to provide you with emotional support?) and social networks (eg, marital status, contact with friends/relatives, membership in religious organization, activities in voluntary groups) *https://pubmed.ncbi.nlm.nih.gov/1443968/*

Williams et al., 1992	18	Perceived socialsupport using structural (eg, marital status) and functional (eg, satisfactionwith social relationships, feeling of loneliness) aspects *https://pubmed.ncbi.nlm.nih.gov/1729574/*

Berkman et al., 2003	19	Low perceived social support determined using the Enhancing Recovery in Coronary Heart Disease Patients (ENRICHED) Social Support Instrument (ESSI) *https://pubmed.ncbi.nlm.nih.gov/12813116/*

**SOCIAL COHESION**		

Kim et al., 2014	20	Perceived neighborhood social cohesion quantified using a four-item scale: (1) I really feel part of this area; (2) If I were in trouble, there are lots of people in this area who would help me; (3) Most people in this area can be trusted; (4) Most people in this area are friendly. *https://pubmed.ncbi.nlm.nih.gov/25135074/*

Lagisetty et al., 2016	21	Perceived neighborhood social cohesionusing five-item Likert scale: (1) People around here arewilling to help their neighbors; (2) People in this neighborhoodgenerally don’t get along with each other; (3) Peoplein this neighborhood can be trusted; (4) People in thisneighborhood do not share the same values; (5) Most peoplein this neighborhood know each other. *https://pubmed.ncbi.nlm.nih.gov/26527589/*

Quinn et al., 2017	22	Neighborhood social cohesion quantified using four questions modified from the Project on Human Development in Chicago Neighborhoods Community Survey *https://www.cdc.gov/pcd/issues/2019/19_0085.htm*

Buckner, 1988	23	Neighborhood Cohesion Instrument(*https://onlinelibrary.wiley.com/doi/abs/10.1007/BF00930892*)

Sampson etal., 1997	24	Social Cohesion Scale *https://science.sciencemag.org/content/277/5328/918*

Smith et al., 2017 Health Retirement Survey	25	Multiple items/subdomains *https://hrs.isr.umich.edu/sites/default/files/biblio/HRS%202006-2016%20SAQ%20Documentation_07.06.17_0.pdf*

**DISCRIMINATION**		

Everson-Rose etal., 2015	26	Discrimination measured using (1) lifetime discrimination with the Lifetime Discrimination Scale and (2) everyday discrimination/with the Everyday Discrimination Scale *https://pubmed.ncbi.nlm.nih.gov/26085044*

Forde et al., 2020	27	Discrimination measured using (1) lifetime discrimination with the Lifetime Discrimination Scale and (2) everyday discrimination using the Everyday Discrimination Scale *https://pubmed.ncbi.nlm.nih.gov/32605388/*

Schulman et al., 1999	28	Discrimination measured as differences inmanagement of chest pain based on race and sex ofpatient in scripted interviews *https://pubmed.ncbi.nlm.nih.gov/10029647/*

Popescu et al., 2011	29	Discrimination measured as differences in acute myocardial infarction admissions to revascularization hospitals and high-quality hospitals based on race *https://pubmed.ncbi.nlm.nih.gov/21632492/*

Wang et al., 2009	30	Discrimination measured as differencein incident hypertension, left ventricular hypertrophy, and barriers to healthcarein patients with a history of incarceration vs those withouta history of incarceration *https://pubmed.ncbi.nlm.nih.gov/19364998/*

**COMMUNITY ENGAGEMENT AND CIVIC PARTICIPATION**		

Victor et al., 2018 Resnicow et al., 2005	31,32	Effects of community engagement on CVD risk factors assessed via engagement in barbershops, local churches *https://www.nejm.org/doi/full/10.1056/NEJMoa1717250* *https://content.apa.org/record/2005-07929-001*

Benson et al., 2019	33	Various community engagement practices, including heart-health screenings, community weight-loss interventions, community health challenges, and phone counseling program *https://pubmed.ncbi.nlm.nih.gov/30792949/*

Sidebottom et al., 2018	34	Community engagementusing multiple interventions in a single town to assess forimprovement in CVD risk factors *https://pubmed.ncbi.nlm.nih.gov/29634974/*

Burr et al., 2011	35	Volunteer work assessed as a community engagement activity *https://journals.sagepub.com/doi/abs/10.1177/0898264310388272*

**ADDITIONAL RESOURCES**		

Driscoll A., 2010	36	“The collaboration betweeninstitutions of higher education and their larger communities (local, regional/state, national, global) for the mutually beneficial exchange of knowledgeand resources in a context of partnership and reciprocity” *https://naspa.tandfonline.com/doi/pdf/10.3200/CHNG.40.1.38-41*

CDC 2011	37	“The process of working collaboratively with and through groups of people affiliated by geographic proximity, special interest, or similar situations to address issues affecting the wellbeing of those people” *https://www.atsdr.cdc.gov/communityengagement/index.html*

Ahmad et al., 2010	38	“Community Engagement in Research is a core elementof any research effort involving communities which requires academic membersto become part of the community and community members tobecome part of the research team, thereby creating a uniqueworking and learning environment before, during, and after the research.” *https://www.ncbi.nlm.nih.gov/pmc/articles/PMC2901283/*


### Social Support

#### Context

Social support is a multifaceted construct that encompasses information and resources available to an individual to deal with a wide spectrum of life’s challenges and stressors.^13^ It is often classified as emotional (empathy, love, trust), instrumental (tangible goods), informational (information provided to cope with stressful situations), and appraisal (affirmative support related to self-evaluation).^10^ Social support is built around a bidirectional “positive emotional exchange” between an individual and his/her social networks, with positive effects on health outcomes.^12^

#### Current Evidence

Social support is linked to physical and mental wellbeing, increased ability to cope with stress, and improved self-care and overall health-related quality of life in individuals with CVD.^13,39^ Gallagher and colleagues^14^ found that older adults with high levels of social support were more likely to consult with a health professional for weight gain, adhere to medication, and exercise regularly compared with those with medium or low levels of social support; these pathways improve overall cardiovascular health and survival.

Through multiple pathways, social support has been shown to improve self-care in patients with heart failure.^15^ For example, findings from a study of social support and survival in patients with heart failure found that patients experiencing both lack of social support and medication nonadherence had a 3.5-times increased risk of adverse cardiac events relative to patients with medication adherence and higher social support.^15^ In the same study, the authors reported a mediation effect of medication adherence, highlighting a possible mechanism through which social support may impact cardiovascular health. Similarly, lack of emotional support has been associated with a significantly increased risk of mortality after hospitalization for myocardial infarction (MI).^17^

In a unique 19-year retrospective cohort study of more than 3,000 men and women, Thurston et al.^40^ found nearly twice the increased risk of incident coronary artery disease associated with experiences of loneliness. Further, it has been reported that individuals without a spouse or close confidant have lower survival rates compared with those who were married, have a confidant, or both.^18^

Despite the evidence documenting a protective effect of social support on cardiovascular health, relatively few studies have evaluated the effectiveness of social support interventions in the context of CVD. In the Enhancing Recovery in Coronary Heart Disease (ENRICHD) trial—the largest study of social support interventions in CVD patients to date—Lett and colleagues^41^ demonstrated that higher levels of perceived social support with cognitive behavioral therapy were associated with improved cardiac outcomes (time to death and reinfarction), but only in patients without elevated depression, suggesting the relevance of psychological wellbeing to the CSC-CVD association. Greater evidence is needed to improve current understanding of the effectiveness of existing interventions and inform future interventions on a population level.

### Social Cohesion

#### Context

Social cohesion is an important measure of the strength of an individual’s ties to his/her community and is defined by Kawachi and Berkman^42^ as “the extent of connectedness and solidarity among groups in a society.” A cohesive society allows mutual sharing of the community’s collective energy and support system via availability of social capital, which is in turn made available through social networks. Social networks are webs of societal relationships—quantified by their range, density, boundedness, and individuals’ characteristics/homogeneity—that act as antecedents of social support.^43,44^ Social cohesion may protect cardiovascular health through multiple pathways, including improved health behaviors, positive psychological and physical health effects, and improved coping ability.^13,45,46^

#### Current Evidence

Findings from a large, prospective study of > 5,000 participants suggest that neighborhood social cohesion may predict 22% lower risk of MI, independent of sociodemographic and clinical predictors.^20^ These results are corroborated by findings from the Mediators of Atherosclerosis in South Asians Living in America (MASALA) Study, which showed nearly 50% lower odds of hypertension associated with high neighborhood cohesion.^21^

Berkman and colleagues^44^ posit that social networks influence health behaviors and, ultimately, health outcomes by providing social support, influencing social engagement/attachment, and increasing access to material goods and resources. In their study of > 2,700 participants from the Framingham Heart Study, Strully et al.^47^ demonstrated that men had nearly 50% higher odds of taking aspirin if a male friend had also been recently taking aspirin; furthermore, women were nearly three times as likely to take aspirin if a female friend recently experienced a cardiovascular event. Similarly, using data for > 23,000 adults from the National Health Interview Survey, Quinn and colleagues^22^ reported that higher social cohesion was associated with 22%, 13% and 14% increased odds of meeting aerobic guidelines, strength guidelines, and combined aerobic and strength guidelines, respectively.

Social isolation has been shown to be a strong risk factor for CVD. A meta-analysis of 16 longitudinal studies found that poor social relationships were associated with 29% increased risk of coronary heart disease and 32% increased risk of stroke.^48^ Prior evidence suggests that socially isolated individuals may experience higher rates of smoking and obesity and are less likely to be physically active relative to those with stronger social bonds.^48,49^ In addition, social isolation and loneliness may lead to chronic stress, which in turn contributes to CVD.^50^ In one of the largest reported prospective studies of social network in CVD, Kawachi and colleagues^16^ followed 32,624 male health professionals over a 4-year period and found that those who were socially isolated had a 90% increased risk for cardiovascular mortality and 121% increased risk of incident stroke compared with those with the highest level of social networks.

Poor social networks/lack of social cohesion may have disproportionate effects on disadvantaged populations, including racial/ethnic minorities. For example, findings from a diverse prospective study of > 5,000 adults suggest that the effects of neighborhood segregation were more prominent in non-Hispanic Blacks (NHBs) than non-Hispanic Whites (NHWs), while no effects were observed in Hispanics.^51^ Conversely, increasing neighborhood social cohesion is associated with a corresponding decrease in interleukin-6 (IL-6) levels, with the strongest association reported in the NHB population (15-point decrease per unit increase in social cohesion).^3,52^ While there is considerable variation in the measurement and operationalization of social cohesion, widely used and validated scales such as the Neighborhood Cohesion Instrument.^23^ Social Cohesion Scale,^24^ and the psychosocial and lifestyle questionnaire from the Health Retirement Survey^25^ assess various aspects of trust, type/strength of social bonds (eg, friendships, exchange of resources), perceived helpfulness/practical help, common values, loyalty, and tolerance (***Table 1***).

### Discrimination

#### Context

The Institute of Medicine defined discrimination as “differences in care that result from biases, prejudices, stereotyping, and uncertainty in clinical communication and decision making.”^53^ While there are multiple forms of discrimination related to race, gender, weight, national origin, religion, and other sociodemographic factors, this review focuses on racial/ethnic discrimination. Most population-level racial/ethnic disparities are linked to structural or institutional racism, which manifests as disparities in employment opportunities, residential segregation, and access to material resources, among others.^54^ In turn, such differential treatment^55^ restricts access to health care and affects quality of care for disadvantaged populations.

Major mechanisms of the discrimination-CVD association include internalized racism and adverse psychological effects, unhealthy coping behaviors, and cumulative psychological and physiological effects of acute and chronic stress.^13,56^ In addition, insufficient cultural competence training and implicit provider bias toward racial/ethnic minorities increases the risk of bias in clinical decision making and affects the quality of the physician-patient relationship, with implications for patients’ trust in the healthcare system.^57,58^

#### Current Evidence

A large population-based study of > 6,000 adults (The Multi-Ethnic Study of Atherosclerosis) found that during a median follow-up of over 10 years, lifetime discrimination experience in two or more domains predicted a 6% to 28% increased risk of CVD.^26^ Similarly, during a 13-year follow-up of participants from the Jackson Heart Study, Forde and colleagues^27^ found that lifetime discrimination was associated with a 50% increased risk of hypertension.

Institutional racism contributes to disparities in both healthcare access and quality.^56^ Existing evidence suggests that racial/ethnic minorities receive lower quality of care compared to NHWs.^59^ For example, it has been previously documented that NHBs with hypertension are less likely to receive psychosocial support and rapport-building statements from physicians and more likely to experience shorter clinic visits compared with their NHW counterparts with similar CVD risk profiles.^60^ In turn, such differential treatment can create gaps in physician-patient communication and compromise the overall quality of care.^56^

Provider-level disparities in adherence to clinical guidelines, medication prescribing, and use of invasive therapies based on patients’ race/ethnicity have been noted in prior studies.^59^ A survey-based study of > 700 physicians found that providers were less likely to refer NHB patients to the cardiac catheterization laboratory compared with NHW patients.^28^ Similarly, NHB patients with MI are less likely to be admitted to facilities with resources for revascularization procedures.^29^ In addition, NHBs who are taken to the catheterization lab have lower odds of door-to-balloon time < 90 minutes and longer revascularization times compared with NHWs.^61^

Unfortunately, knowledge of discrimination in health care and its resulting disparities is still low among cardiologists. Findings from a web-based survey of nearly 350 cardiologists found that only one-third of providers agreed that racial disparities existed in cardiac care, merely 12% felt that it was present in their institution, and just 5% felt that their patients were affected by it. Interestingly, physicians caring for NHB and Hispanic patients had an even lower perception of the existence of healthcare disparities.^62^ Feelings of implicit bias and provider discrimination among the NHB population have been documented to lower their trust in the healthcare system, leading to missed doctor appointments.^63^

Discrimination is a strong correlate of health and wellness among those who are incarcerated. CVD is the second-leading cause of death in the incarcerated^64^ population, with a disproportionate impact on racial/ethnic minority populations. Prior evidence suggests worse CVD outcomes in the incarcerated population relative to the nonincarcerated and higher CVD risk in NHBs compared with NHWs.^30^ However, current knowledge of the long-term impact of incarceration on the cardiovascular health of racial/ethnic minorities is limited and mandates further study.

### Community Engagement and Civic Participation

#### Context

Community engagement encourages community members to plan, design, and implement public health interventions and is an established tool to reduce disparities and inequities in health and health care.^65^ The concept of civic participation means participating in a variety of community-level activities that foster societal relationships, strengthen social bonds and networks, and improve health and wellbeing—both on individual and community levels.^66^ Both community engagement and civic participation have beneficial effects on cardiovascular health.

#### Current Evidence

In the Community Outreach and Cardiovascular Health (COACH) trial, patients with CVD, type 2 diabetes, or hypercholesterolemia were randomized to either enhanced usual care (control arm) or to the intervention arm, which included CVD risk factor management with a nurse practitioner/community health worker.^67^ The intervention group had significantly higher improvements in total cholesterol, LDL cholesterol, triglycerides, systolic and diastolic blood pressures, and hemoglobin A1c.

The HONU (Heart of New Ulm) is a population-level CVD prevention project that engages a variety of community stakeholders to reduce CVD risk in the community through heart-health screenings, community weight-loss interventions, community health challenges, and a phone counseling program for high-risk residents. The project’s multipronged approach to community engagement over the course of 5 years yielded a significant improvement in a variety of CVD risk factors, including physical activity and daily fruit and vegetable intake.^33^ Compared with matched controls from a similar community over the span of 7 years, the community at New Ulm had higher rates of blood pressure control, lower triglyceride levels, higher medication compliance (lipid medication and aspirin), and smaller increases in atherosclerotic CVD risk scores.^31^

Health advocacy by barbers, coupled with medication management by pharmacists, has been shown to be helpful in improving health behaviors in the NHB community.^31^ In a cohort of 319 NHB males with systolic blood pressure (SBP) ≥ 140 mm Hg, 139 barbershop patrons were assigned to an intervention involving medication management by a pharmacist in the shop (cases) and 180 patrons received lifestyle modification tips and encouragement to set up doctor appointments (controls). At the end of 6 months, mean SBP dropped by 27 mm Hg in cases compared with 9.3 mm Hg in the control group.^31,34^

Local churches have also been successful in improving community health behaviors. Findings from the Healthy Body Healthy Spirit trial of > 1,000 individuals recruited across 16 churches showed that a combination of standard educational materials, nutritional/physical activity resources, and motivational interviewing (via telephone counseling calls) significantly increased both fruit and vegetable consumption and physical activity.^32^

Civic participation, such as volunteering, voting, and a variety of group recreational and sporting activities (eg, hockey, soccer, gardening, cleaning, etc.), strengthens existing social networks, increases social cohesion, creates a common sense of goals and purpose, and improves overall health and wellbeing.^68^ A study of > 7,000 middle-aged and older adults found that greater participation in volunteering activities was associated with 22% lower odds of central adiposity and 26% lower odds of lipid dysregulation. Similarly, another study of > 5,600 middle-aged and older men and women documented a 20% lower risk of hypertension and lower blood pressure levels overall among individuals who reported volunteering.^35^ Civic participation may also improve overall CVD risk profile by improving physical activity and expanding/strengthening social networks, as documented in a study of Hispanic individuals that found that increased civic participation promoted physical activity, regardless of the size of social networks and awareness of physical activity resources.^69^

## Pathways from CSC to CVD

**Figure 2:**
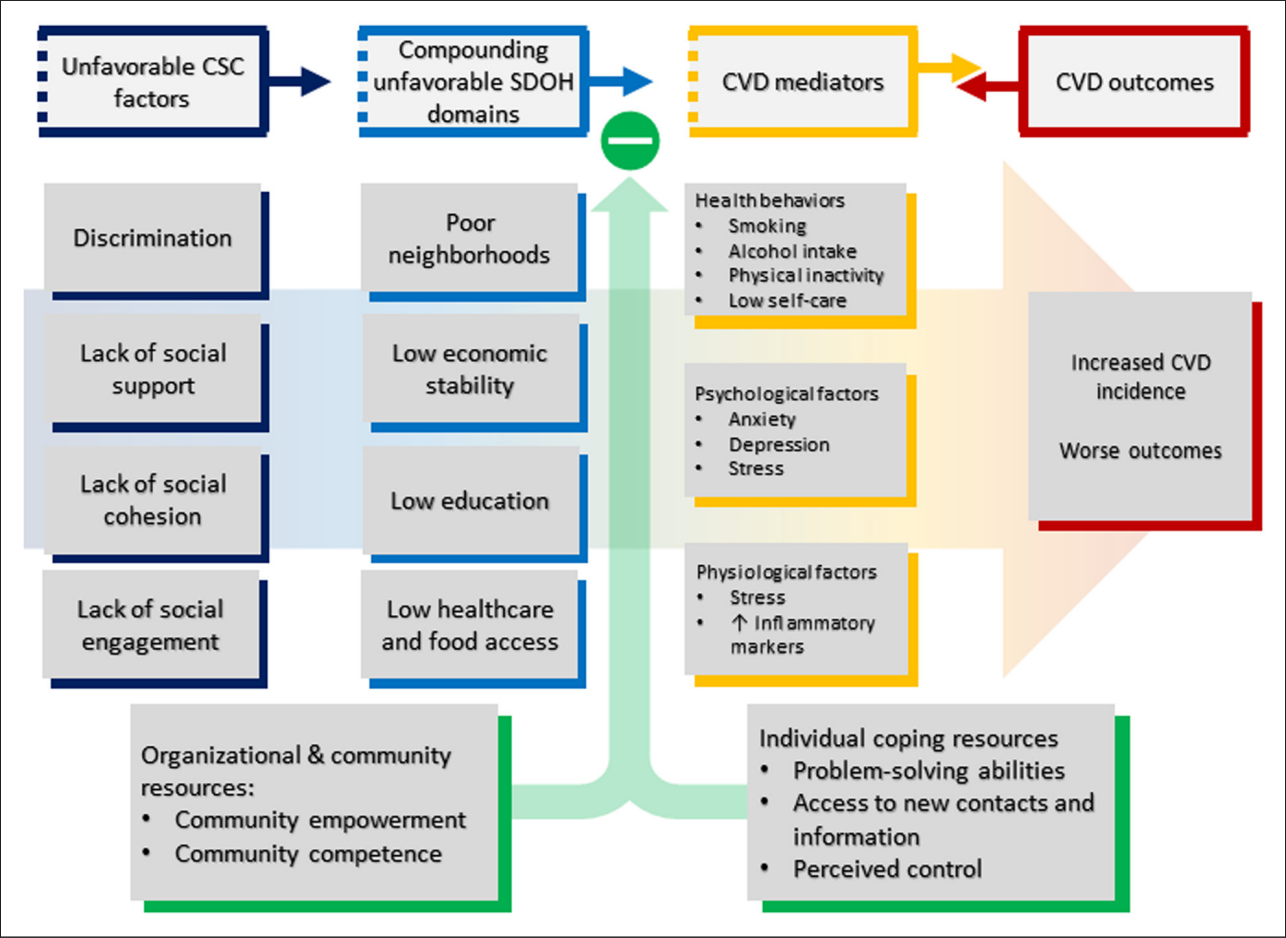
Pathways from community and social context (CSC) to cardiovascular disease (CVD). SDOH: social determinants of health.

The theoretical foundations of social support and all four subconstructs are grounded in the social comparison, social exchange, and social competence theories.^70^ The positive impact of each type of support is facilitated by social networks, social cohesion/community engagement, and the overall psychosocial climate of an individual’s environment.^70^ These pathways are summarized in ***Figure 2***.

It is posited that a positive psychosocial climate, including attributes of helpfulness and protection, helps develop social competence, which in turn positively reinforces self-esteem, psychological wellbeing, and the ability to cope with stress.^70,71^ Social competence further enhances the positive, bidirectional, mutually rewarding association between an individual and the networks that provide social support, ensuring overall “social health”—an important determinant of psychological wellbeing.^70,72^

Social support and associated constructs influence health outcomes via both physiological and psychological stress response as well as health behaviors.^44^ Lack of social cohesion and trust have been associated with poor mental health outcomes, and limited social support or weak/small social networks—largely prevalent among disadvantaged groups—are associated with negative emotional states.^73^ Similarly, the effects of poor social support and/or community engagement might be mediated by poor health behaviors, such as smoking, excessive alcohol consumption, and low physical activity levels.^74^

The psychological and behavioral responses to unfavorable community/social exposures potentiate harmful physiological responses, such as activated hypothalamic-pituitary-adrenal (HPA) axis and raised levels of inflammatory markers.^75,76^ For example, acute stress is documented to be associated with raised IL-6 levels in women with low self-reported social support.^76^ Social isolation and low social support are linked to increased heart rate, blood pressure, and cortisol levels in preclinical studies.^77,78^ Similarly, poor social support is linked to increased HPA axis reactivity and associated effects, such as increased heart rate and blood pressure.^79^

Major mechanisms of the discrimination-CVD association include internalized racism and adverse psychological effects (negative emotional state, heightened anticipatory vigilance, psychological distress, etc.), unhealthy coping behaviors, and cumulative psychological and physiological effects of acute and chronic stress.^13,54,56^ These contribute to elevated blood pressure, decreased insulin sensitivity, and increased coronary artery calcium.^80,81^ Additional factors at the healthcare level include lack of cultural competence training and implicit provider bias toward racial/ethnic minorities and other disadvantaged population subgroups, with implications for quality of care for marginalized populations and patient trust in the healthcare system.^57,58^

Additional evidence is needed to understand potential intersectionality among different CSC subdomains. Future studies should also assess how CSC effects are potentially modified via socioeconomic and demographic pathways.

## Conclusions

Community and social context affect cardiovascular health via multiple subdomains and diverse pathways. Social support, social cohesion, discrimination, and community engagement and civic participation uniquely determine social networks and social capital, ability to seek and/or provide help, ability to cope with stress, neighborhood trust and strength of social bonds, bias and prejudice, and overall sense of goals and common purpose. In turn, these and related CSC factors shape one’s susceptibility to illness and access to helpful resources, thereby determining individual-, community-, and population-level health outcomes.

The effects of individual CSC subdomains manifest via both upstream (eg, material resources, access/quality of care) and downstream (eg, unhealthy coping behaviors) factors. These constructs impact CVD risk via multiple physiologic, psychosocial, and emotional pathways, including the role of stress as a mediator of increased CVD risk and poor disease outcomes.

The findings of this review are intended to increase awareness of the impact of social and environmental conditions on cardiovascular health and serve as a resource for healthcare providers and health equity champions, both on practice and policy levels. Given the country’s current social and political climate, we are confident that the evidence presented herein will stimulate future discussion on addressing CSC-related inequities in CVD morbidity and mortality, with particular implications for socially disadvantaged communities.

Key recommendations to address major knowledge gaps in the field and advance current understanding of the pathways, mechanisms, and overall effects of CSC were presented in ***Table 2***. Future efforts should focus on developing strategies to incorporate CSC into clinical risk-prediction algorithms and informing CVD prevention and management guidelines and practices.

**Table 2:** Subdomain-specific research and policy recommendations. CSC: community and social context; CVD: cardiovascular disease; SDOH: social determinants of health.


CSC SUBDOMAINS	RECOMMENDATIONS FOR FUTURE WORK

**SOCIAL SUPPORT**	Conduct large-scale population-based studies to further elucidate pathways from CSC to CVD. Inform community-level social support interventions using evidence from both observational and experimental studies. Increase focus on social support-CVD link in disadvantaged populations, including racial/ethnic minorities. Develop validated, generalizable measures of social support.

**SOCIAL COHESION**	Future study should focus on increasing understanding of potential moderating effects of race/ethnicity on the social cohesion-CVD relationship. Future research should improve understanding of pathways linking social cohesion/networks and CVD, including the role of health behaviors and psychological wellbeing. Investigate possible intersectional effects of race/ethnicity and other SDOH, on CVD outcomes.

**DISCRIMINATION**	Define and develop tools to measure/analyze discrimination and bias in health care. Elucidate major physiologic, psychological, and behavioral pathways from perceived discrimination to CVD. Improve current understanding of the effects of internalized racism and health behaviors in marginalized populations. Develop evidence-based interventions to address health system factors contributing to racial/ethnic disparities in CVD, such as implicit bias and lack of cultural competence.

**COMMUNITY ENGAGEMENT AND CIVIC PARTICIPATION**	Design and implement community-level CVD prevention interventions: identify community leaders and engage relevant stakeholders. Document potential variation in civic participation by different sociodemographic factors, including sex and race/ethnicity. Describe pathways linking civic participation to improved CVD outcomes. Increase representation and participation of underserved communities in community-based CVD prevention programs.


## Key Points

- Individual and societal relationships are key determinants of health and wellbeing, and high social cohesion is documented to have a strong protective effect on cardiovascular health. Conversely, poor social bonds and weak social networks predict poor cardiovascular health, with a disproportionate impact on vulnerable communities.
- Evidence for a positive effect of social support on cardiovascular disease (CVD) outcomes—including the long-term impact of social support interventions—is lacking and merits greater research, as does evidence to develop a standardized social support measurement tool.
- Racial/ethnic discrimination is linked to both cardiovascular risk factors and adverse CVD outcomes, including hypertension, stroke, and coronary heart disease. Although various pathways explain the link between discrimination and CVD, existing understanding is limited and merits further study.
- Current evidence suggests that community engagement and civic participation promote positive behavioral changes, strengthen social bonds/networks, and exert a protective effect on cardiovascular health.
- Greater civic engagement and representation of marginalized populations in community engagement initiatives is essential to maximizing the benefits of such interventions and improving health outcomes on a population level.
- Medical training must acknowledge and address issues such as cultural competence with the aim of reducing implicit provider bias in clinical decision making.

## References

[1] Virani SS, Alonso A, Benjamin EJ, et al. Heart Disease and Stroke Statistics-2020 Update: A Report From the American Heart Association. Circulation. 2020 Mar 3;141(9):e139–e596. doi: 10.1161/CIR.0000000000000757 31992061

[2] CDC.gov [Internet]. Atlanta, GA: Centers for Disease Control and Prevention; c2021. Heart Disease facts; 2020 Sep 8 [cited 2021 Sep 3]. Available from: https://www.cdc.gov/heartdisease/facts.htm#:~:text=Heart%20disease%20is%20the%20leading,1%20in%20every%204%20deaths

[3] Rubinstein A, Colantonio L, Bardach A, et al. Estimation of the burden of cardiovascular disease attributable to modifiable risk factors and cost-effectiveness analysis of preventative interventions to reduce this burden in Argentina. BMC Public Health. 2010 Oct 20;10:627. doi: 10.1186/1471-2458-10-627 20961456 PMC2970607

[4] WHO.int [Internet]. Geneva, Switzerland: World Health Organization; c2021. Global status report on noncommunicable diseases 2014; 2014 Oct 26 [cited 2021 Sep 2]. Available from: https://www.who.int/publications/i/item/9789241564854

[5] Angermayr L, Melchart D, Linde K. Multifactorial lifestyle interventions in the primary and secondary prevention of cardiovascular disease and type 2 diabetes mellitus--a systematic review of randomized controlled trials. Ann Behav Med. 2010;40(1):49–64. 20652464 10.1007/s12160-010-9206-4

[6] Arnett DK, Blumenthal RS, Albert MA, et al. 2019 ACC/AHA guideline on the primary prevention of cardiovascular disease: a report of the American College of Cardiology/American Heart Association Task Force on Clinical Practice Guidelines. Circulation; 2019 Sep 10;140(11):e596–e646. doi: 10.1161/CIR.0000000000000678 30879355 PMC7734661

[7] CDC.gov [Internet]. Atlanta, GA: Centers for Disease Control and Prevention; c2021. State Heart Disease and Stroke Prevention Program in Health Care Settings to Prevent Heart Disease and Stroke; 2014 Jul 22 [cited 2021 Sept 3]. Available from: https://www.cdc.gov/dhdsp/data_statistics/fact_sheets/fs_state_healthcare.htm

[8] Health.gov [Internet]. Washington, DC: US Department of Health and Human Services; c 2021. HealthyPeople 2030: Social Determinants of Health; 2021 [cited 2021 Sep 3]. Available from: https://health.gov/healthypeople/objectives-and-data/social-determinants-health

[9] Reblin M, Uchino BN. Social and emotional support and its implication for health. Curr Opin Psychiatry. 2008 Mar;21(2):201–5. doi: 10.1097/YCO.0b013e3282f3ad89 18332671 PMC2729718

[10] KFF.org [Kaiser Family Foundation]. San Francisco, CA: Kaiser Family Foundation; c2021. Beyond Health Care: The Role of Social Determinants in Promoting Health and Health Equity; 2018 May 10 [cited 2021 Sep 3]. Available from: https://www.kff.org/racial-equity-and-health-policy/issue-brief/beyond-health-care-the-role-of-social-determinants-in-promoting-health-and-health-equity/

[11] CDC.gov [Internet]. Atlanta, GA: Centers for Disease Control and Prevention; c2021. Social Determinants of Health. Know What Affects Health; 2021 May 6 [cited 2021 Sep 3]. Available from: https://www.cdc.gov/socialdeterminants/index.htm

[12] HealthyPeople.gov [Internet]. Washington, DC: US Department of Health and Human Services; c2021. Social Determinants of Health: Explore Resources Related to the Social Determinants of Health; 2021 [cited 2021 Sep 3]. Available from: https://www.healthypeople.gov/2020/topics-objectives/topic/social-determinants-health/interventions-resources

[13] Havranek EP, Mujahid MS, Barr DA, et al. Social Determinants of Risk and Outcomes for Cardiovascular Disease: A Scientific Statement From the American Heart Association. Circulation. 2015 Sep 1;132(9):873–98. doi: 10.1161/CIR.0000000000000228 26240271

[14] Gallagher R, Luttik M-L, Jaarsma T. Social support and self-care in heart failure. J Cardiovasc Nurs. Nov-Dec 2011;26(6):439–45. doi: 10.1097/JCN.0b013e31820984e1 21372734

[15] Wu JR, Frazier SK, Rayens MK, Lennie TA, Chung ML, Moser DK. Medication adherence, social support, and event-free survival in patients with heart failure. Health Psychol. 2013 Jun;32(6):637–46. doi: 10.1037/a0028527 22746258 PMC4057061

[16] Kawachi I, Colditz GA, Ascherio A, et al. A prospective study of social networks in relation to total mortality and cardiovascular disease in men in the USA. J Epidemiol Community Health. 1996 Jun;50(3):245–51. doi: 10.1136/jech.50.3.245 8935453 PMC1060278

[17] Berkman LF, Leo-Summers L, Horwitz RI. Emotional support and survival after myocardial infarction: a prospective, population-based study of the elderly. Ann Intern Med. 1992 Dec 15;117(12):1003–9. doi: 10.7326/0003-4819-117-12-1003 1443968

[18] Williams RB, Barefoot JC, Califf RM, et al. Prognostic importance of social and economic resources among medically treated patients with angiographically documented coronary artery disease. JAMA. 1992 Jan 22-29;267(4): 520–4. 1729574

[19] Berkman LF, Blumenthal J, Burg M, et al. Effects of treating depression and low perceived social support on clinical events after myocardial infarction: the Enhancing Recovery in Coronary Heart Disease Patients (ENRICHD) Randomized Trial. JAMA. 2003 Jun 18;289(23):3106–16. doi: 10.1001/jama.289.23.3106 12813116

[20] Kim ES, Hawes AM, Smith J. Perceived neighbourhood social cohesion and myocardial infarction. J Epidemiol Community Health. 2014 Nov;68(11):1020–26. 25135074 10.1136/jech-2014-204009 PMC4600604

[21] Lagisetty PA, Wen M, Choi H, Heisler M, Kanaya AM, Kandula NR. Neighborhood social cohesion and prevalence of hypertension and diabetes in a South Asian population. J Immigr Minor Health. 2016 Dec;18(6):1309–16. doi: 10.1007/s10903-015-0308-8 26527589 PMC4853276

[22] Quinn TD, Wu F, Mody D, et al. Associations Between Neighborhood Social Cohesion and Physical Activity in the United States, National Health Interview Survey, 2017. Preventing chronic disease. 2019 Dec 19;16. doi: 10.5888/pcd16.190085 PMC6936668 31858956

[23] Buckner JC. The development of an instrument to measure neighborhood cohesion. Am J Comm Psych. 1988;16(6):771–791.

[24] Sampson RJ, Raudenbush SW, Earls F. Neighborhoods and violent crime: A multilevel study of collective efficacy. Science. 1997 Aug 15;277(5328):918–24. doi: 10.1126/science.277.5328.918 9252316

[25] Smith J, Ryan L, Sonnega A, Weir D. Psychosocial and lifestyle questionnaire 2006–2016. Ann Arbor, MI: Survey Research Center, Institute for Social Research. 2017 Jul.

[26] Everson-Rose SA, Lutsey PL, Roetker NS, et al. Perceived Discrimination and Incident Cardiovascular Events: The Multi-Ethnic Study of Atherosclerosis. Am J Epidemiol. 2015 Aug 1;182(3):225–34. doi: 10.1093/aje/kwv035 26085044 PMC4517694

[27] Forde AT, Sims M, Muntner P, et al. Discrimination and hypertension risk among African Americans in the Jackson heart study. Hypertension. 2020 Sep;76(3):715–723. doi: 10.1161/HYPERTENSIONAHA.119.14492 32605388 PMC8359680

[28] Schulman KA, Berlin JA, Harless W, et al. The effect of race and sex on physicians’ recommendations for cardiac catheterization. N Engl J Med. 1999 Feb 25;340(8):618–26. doi: 10.1056/NEJM199902253400806 10029647

[29] Popescu I, Cram P, Vaughan-Sarrazin MS. Differences in admitting hospital characteristics for black and white Medicare beneficiaries with acute myocardial infarction. Circulation. 2011 Jun 14;123(23):2710–6. doi: 10.1161/CIRCULATIONAHA.110.973628 21632492 PMC3142883

[30] Wang EA, Pletcher M, Lin F, et al. Incarceration, incident hypertension, and access to health care: findings from the coronary artery risk development in young adults (CARDIA) study. Arch Intern Med. 2009 Apr 13;169(7):687–93. doi: 10.1001/archinternmed.2009.26 19364998 PMC2829673

[31] Victor RG, Lynch K, Li N, et al. A Cluster-Randomized Trial of Blood-Pressure Reduction in Black Barbershops. N Engl J Med. 2018 Apr 5;378(14):1291–1301. doi: 10.1056/NEJMoa1717250 29527973 PMC6018053

[32] Resnicow K, Jackson A, Blissett D, et al. Results of the healthy body healthy spirit trial. Health Psychol. 2005 Jul;24(4):339–48. doi: 10.1037/0278-6133.24.4.339 16045368

[33] Benson G, Sidebottom AC, Sillah A, et al. Population-level changes in lifestyle risk factors for cardiovascular disease in the Heart of New Ulm Project. Prev Med Rep. 2019 Jan 31;13:332–340. doi: 10.1016/j.pmedr.2019.01.018 30792949 PMC6369314

[34] Sidebottom AC, Sillah A, Vock DM, et al. Assessing the impact of the heart of New Ulm Project on cardiovascular disease risk factors: A population-based program to reduce cardiovascular disease. Prev Med. 2018 Jul;112:216–221. doi: 10.1016/j.ypmed.2018.04.016 29634974

[35] Burr JA, Tavares J, Mutchler JE. Volunteering and hypertension risk in later life. J Aging Health. 2011 Feb;23(1):24–51. doi: 10.1177/0898264310388272 20971920

[36] Driscoll A. Carnegie’s community-engagement classification: Intentions and insights. Change: The Magazine of Higher Learning. 2008;40(1):38–41.

[37] CDC.gov [Internet]. Atlanta, GA: Centers for Disease Control and Prevention; c2021. Agency for Toxic Substances and Disease Registry: Principles of Community Engagement - Second Edition; 2015 Jun 25 [cited 2021 Sep 3]. Available from: https://www.atsdr.cdc.gov/communityengagement/index.html

[38] Ahmed SM, Palermo A-G S. Community engagement in research: frameworks for education and peer review. Am J Public Health. 2010 August;100(8):1380–7. doi: 10.2105/AJPH.2009.178137 20558798 PMC2901283

[39] Cobb S. Presidential Address—1976. Social support as a moderator of life stress. Psychosom Med. Sep-Oct 1976;38(5):300–14. doi: 10.1097/00006842-197609000-00003 981490

[40] Thurston RC, Kubzansky LD. Women, loneliness, and incident coronary heart disease. Psychosom Med. 2009 Oct;71(8):836–42. doi: 10.1097/PSY.0b013e3181b40efc 19661189 PMC2851545

[41] ClinicalTrials.gov [Internet]. Bethesda, MD: US National Library of Medicine; c 2021. Enhancing Recovery in Coronary Heart Disease (ENRICHD) Patients; 2016 Apr 14 [cited 2021 Sep 3]. Available from: https://clinicaltrials.gov/ct2/show/study/NCT00000557

[42] Kawachi I, Berkman L. Social cohesion, social capital, and health. In: Berkman LF, Kawachi I, editors. Social Epidemiology. New York, NY: Oxford University Press; 2000. p. 147–190.

[43] HealthyPeople.gov [Internet]. Washington, DC: US Department of Health and Human Services; c2021. Social Cohesion; 2021 [cited 2021 Sep 3]. Available from: https://www.healthypeople.gov/2020/topics-objectives/topic/social-determinants-health/interventions-resources/social-cohesion

[44] Berkman LF, Glass T, Brissette I, Seeman TE. From social integration to health: Durkheim in the new millennium. Soc Sci Med. 2000 Sep;51(6):843–57. doi: 10.1016/s0277-9536(00)00065-4 10972429

[45] Choi YJ, Matz-Costa C. Perceived neighborhood safety, social cohesion, and psychological health of older adults. Gerontologist. 2018 Jan 18;58(1):196–206. doi: 10.1093/geront/gnw187 28082279

[46] Fan C, Jiang Y, Mostafavi A. Emergent social cohesion for coping with community disruptions in disasters. J R Soc Interface. 2020 Mar;17(164):20190778. doi: 10.1098/rsif.2019.0778 32126194 PMC7115229

[47] Strully KW, Fowler JH, Murabito JM, Benjamin EJ, Levy D, Christakis NA. Aspirin use and cardiovascular events in social networks. Soc Sci Med. 2012 Apr;74(7):1125–9. doi: 10.1016/j.socscimed.2011.12.033 22361089 PMC3298609

[48] Lauder W, Mummery K, Jones M, Caperchione C. A comparison of health behaviours in lonely and non-lonely populations. Psychol Health Med. 2006 May;11(2):233–245. doi: 10.1080/13548500500266607 17129911

[49] Shelton RC, McNeill LH, Puleo E, Wolin KY, Emmons KM, Bennett GG. The association between social factors and physical activity among low-income adults living in public housing. Am J Public Health. 2011 Nov;101(11):2102–2110. doi: 10.2105/AJPH.2010.196030 21330588 PMC3193546

[50] Valtorta NK, Kanaan M, Gilbody S, Ronzi S, Hanratty B. Loneliness and social isolation as risk factors for coronary heart disease and stroke: systematic review and meta-analysis of longitudinal observational studies. Heart. 2016 Jul 1;102(13):1009–16. doi: 10.1136/heartjnl-2015-308790 27091846 PMC4941172

[51] Kershaw KN, Osypuk TL, Do DP, De Chavez PJ, Diez Roux AV. Neighborhood-level racial/ethnic residential segregation and incident cardiovascular disease: the multi-ethnic study of atherosclerosis. Circulation. 2015 Jan 13;131(2):141–8. doi: 10.1161/CIRCULATIONAHA.114.011345 25447044 PMC4293329

[52] Neergheen VL, Topel M, Van Dyke ME, et al. Neighborhood social cohesion is associated with lower levels of interleukin-6 in African American women. Brain Behav Immun. 2019 Feb;76:28–36. doi: 10.1016/j.bbi.2018.10.008 30686334 PMC6370481

[53] Smedley BD, Stith AY, Nelson AR, editor. Unequal Treatment: Confronting Racial and Ethnic Disparities in Health Care. Washington, DC: National Academies Press; c2003. doi: 10.17226/12875 25032386

[54] Williams DR, Mohammed SA. Discrimination and racial disparities in health: evidence and needed research. J Behav Med. 2009 Feb;32(1):20–47. doi: 10.1007/s10865-008-9185-0 19030981 PMC2821669

[55] Yearby R. The impact of structural racism in employment and wages on minority women’s health. Hum Rts. 2018 Mar 18;43(3):21.

[56] Brewer LC, Cooper LA. Race, discrimination, and cardiovascular disease. Virtual Mentor. 2014 Jun 1;16(6):270–4. doi: 10.1001/virtualmentor.2014.16.06.stas2-1406 24955594

[57] FitzGerald C, Hurst S. Implicit bias in healthcare professionals: a systematic review. BMC Med Ethics. 2017 Mar 1;18(1):19. doi: 10.1186/s12910-017-0179-8 28249596 PMC5333436

[58] Saha S, Beach MC, Cooper LA. Patient centeredness, cultural competence and healthcare quality. J Natl Med Assoc. 2008 Nov;100(11):1275–85. doi: 10.1016/s0027-9684(15)31505-4 19024223 PMC2824588

[59] Mody P, Gupta A, Bikdeli B, Lampropulos JF, Dharmarajan K. Most important articles on cardiovascular disease among racial and ethnic minorities. Circ Cardiovasc Qual Outcomes. 2012 Jul 1;5(4):e33–41. doi: 10.1161/CIRCOUTCOMES.112.967638 22811508

[60] Cené CW, Roter D, Carson KA, Miller ER, Cooper LA. The effect of patient race and blood pressure control on patient-physician communication. J Gen Intern Med. 2009 Sep;24(9):1057–64. doi: 10.1007/s11606-009-1051-4 19575270 PMC2726885

[61] Cavender MA, Rassi AN, Fonarow GC, et al. Relationship of race/ethnicity with door-to-balloon time and mortality in patients undergoing primary percutaneous coronary intervention for ST-elevation myocardial infarction: findings from Get With the Guidelines-Coronary Artery Disease. Clin Cardiol. 2013 Dec;36(12):749–56. doi: 10.1002/clc.22213 24085713 PMC6649362

[62] Lurie N, Fremont A, Jain AK, et al. Racial and ethnic disparities in care: the perspectives of cardiologists. Circulation. 2005 Mar 15;111(10):1264–9. doi: 10.1161/01.CIR.0000157738.12783.71 15769767

[63] Greer TM. Perceived racial discrimination in clinical encounters among African American hypertensive patients. J Health Care Poor Underserved. 2010 Feb;21(1):251–63. doi: 10.1353/hpu.0.0265 20173267

[64] Binswanger IA, Stern MF, Deyo RA, et al. Release from prison—a high risk of death for former inmates. N Engl J Med. 2007 Jan 11;356(2):157–65. doi: 10.1056/NEJMsa064115 17215533 PMC2836121

[65] O’Mara-Eves A, Brunton G, Oliver S, Kavanagh J, Jamal F, Thomas J. The effectiveness of community engagement in public health interventions for disadvantaged groups: a meta-analysis. BMC Public Health. 2015 Feb 12;15:129. doi: 10.1186/s12889-015-1352-y 25885588 PMC4374501

[66] Abbott S. Social capital and health: The role of participation. Soc Theory Health. 2010;8(1):51–65. doi: 10.1057/sth.2009.19

[67] Allen JK, Dennison-Himmelfarb CR, Szanton SL, et al. Community Outreach and Cardiovascular Health (COACH) Trial: a randomized, controlled trial of nurse practitioner/community health worker cardiovascular disease risk reduction in urban community health centers. Circ Cardiovasc Qual Outcomes. 2011 Nov 1;4(6):595–602. doi: 10.1161/CIRCOUTCOMES.111.961573 21953407 PMC3218795

[68] Kim S, Kim C-y, You MS. Civic participation and self-rated health: a cross-national multi-level analysis using the world value survey. J Prev Med Public Health. 2015 Jan;48(1):18–27. doi: 10.3961/jpmph.14.031 25652707 PMC4322515

[69] Marquez B, Gonzalez P, Gallo L, Ji M. Latino Civic Group Participation, Social Networks, and Physical Activity. Am J Health Behav. 2016 Jul;40(4):437–45. doi: 10.5993/AJHB.40.4.5 27338990 PMC8213013

[70] Langford CPH, Bowsher J, Maloney JP, Lillis PP. Social support: a conceptual analysis. J Adv Nurs. 1997 Jan;25(1):95–100. doi: 10.1046/j.1365-2648.1997.1997025095.x 9004016

[71] Integrating Social Support in Nursing Integrating Social Support in Nursing M. j. Stewart SAGE 246pp £12.95 0-8039-4274-5. Nurs Stand. 1994 Jul 20;8(43):40. doi: 10.7748/ns.8.43.40.s57 27669955

[72] Stevens ES. Reciprocity in social support: An advantage for the aging family. Fam Soc. 1992;73(9):533–541. 10.1177/104438949207300903

[73] Wang J, Mann F, Lloyd-Evans B, Ma R, Johnson S. Associations between loneliness and perceived social support and outcomes of mental health problems: a systematic review. BMC Psychiatry. 2018 May 29;18(1):156. doi: 10.1186/s12888-018-1736-5 29843662 PMC5975705

[74] Steptoe A, Kivimäki M. Stress and cardiovascular disease. Nat Rev Cardiol. 2012 Apr 3;9(6):360–70. doi: 10.1038/nrcardio.2012.45 22473079

[75] Uchino BN, Cacioppo JT, Kiecolt-Glaser JK. The relationship between social support and physiological processes: a review with emphasis on underlying mechanisms and implications for health. Psychol Bull. 1996 May;119(3):488–531. doi: 10.1037/0033-2909.119.3.488 8668748

[76] Puterman E, Epel ES, O’Donovan A, Prather AA, Aschbacher K, Dhabhar FS. Anger is associated with increased IL-6 stress reactivity in women, but only among those low in social support. Int J Behav Med. 2014 Dec;21(6):936–45. doi: 10.1007/s12529-013-9368-0 24357433 PMC4406249

[77] Shively CA, Clarkson TB, Kaplan JR. Social deprivation and coronary artery atherosclerosis in female cynomolgus monkeys. Atherosclerosis. 1989 May;77(1):69–76. doi: 10.1016/0021-9150(89)90011-7 2719764

[78] Sapolsky RM, Alberts SC, Altmann J. Hypercortisolism associated with social subordinance or social isolation among wild baboons. Arch Gen Psychiatry. 1997 Dec;54(12):1137–43. doi: 10.1001/archpsyc.1997.01830240097014 9400351

[79] Steptoe A, Owen N, Kunz-Ebrecht SR, Brydon L. Loneliness and neuroendocrine, cardiovascular, and inflammatory stress responses in middle-aged men and women. Psychoneuroendocrinology. 2004 Jun;29(5):593–611. doi: 10.1016/S0306-4530(03)00086-6 15041083

[80] Brotman DJ, Golden SH, Wittstein IS. The cardiovascular toll of stress. Lancet. 2007 Sep 22;370(9592):1089–100. doi: 10.1016/S0140-6736(07)61305-1 17822755

[81] Panza GA, Puhl RM, Taylor BA, Zaleski AL, Livingston J, Pescatello LS. Links between discrimination and cardiovascular health among socially stigmatized groups: A systematic review. PLoS One. 2019 Jun 10;14(6):e0217623. doi: 10.1371/journal.pone.0217623 31181102 PMC6557496

